# Our New York Letter

**Published:** 1892-08

**Authors:** 


### Correspondence.

### NEW YORK LETTER.

NEW YORK, July 19, 1892.

Pyoktanin is now largely employed by gynecologists in this city in the treatment of carcinoma of the uterus, and with very marked success. In treating these cases by means of this drug, the uterus is first thoroughly curetted and packed with iodoform gauze. The gauze is removed after twenty-four hours, and a 1-300 solution of pyoktanin is then injected. Dr. Boldt, of this city, has at present thirteen cases of carcimona of the uterus under treatment by this method. In all cases radical treatment by hysterectomy was absolutely contra-indicated. The parovarium was infiltrated, and in six of the cases the vagina was involved, and in one case the disease had extended to the bladder. In only two of the thirteen cases did the disease progress, while in the others it remained stationary.

The general opinion of gynecologists in this city appears to be in favor of the use of pyoktanin in carcinoma of the uterus, as in most of the cases treated in this city the patients were greatly benefitted by its use.

The death rate has been extremely high this month, as compared with the same period last year. For the twenty-four hours ending at noon on July 12th, there were 260 deaths in this city, which was the largest number of deaths for any one day reported in over three years. This large death rate was chiefly among children, upon whom the effects of the hot weather are felt with special force.

Dr. DeGarmo, in speaking quite recently on the use of trusses for hernia in children, said that he condemned all infant trusses which are made to apply from the side of the rupture, all where the pad is placed upon a decending arm at a level lower than the pelvic spring, and all trusses padded with soft material. In making a selection of a truss, the following points should be borne in mind: First, the spring should be so tempered that it may be readily bent to the exact shape of the child, and its pressure added to, or diminished, by increasing or removing the amount of curve which it possesses. Second, the entire truss should be impervious to moisture, that it may be frequently washed; and third, it should be durable. The more simple in design a truss is, the better it is, as a rule.

The truss should be worn for at least one year. For the cure of umbilical hernia in infants, he advises the use of a cork as a compress, so cut as to press slightly into the navel and support the surrounding parts. The corks used by druggists for wide mouthed bottles will serve this purpose exceedingly well. A thin layer of absorbent cotton is placed under this, and the whole is strapped down by a one-inch strap of Mead's rubber plaster passing two-thirds around the body. It will be sufficient to change this dressing but once a month. In infants, Dr. DeGarmo has found that the injection of Heaton's fluid helps the cure, though it rarely succeeds with adults.

At a recent clinic held by Dr. Sims, at the New York Polyclinic, a woman 23 years of age, presented with the symptoms of amenorrhœa, due to a non-development of the uterus. Speaking of the treatment of this condition, Dr. Sims said that the only plan he has found to be better than all others, was dilatation of the canal and stimulation of the uterus, and permitting the patient to wear a self-retaining stem for a considerable length of time. In this way, the uterus gradually grows in size, and menstruation appears with more or less regularity, until the courses are completely and permanently established.

Though the colleges are all closed for the summer season, the hospital clinics are still being kept up with unabated interest, and are well attended by doctors from out of town. There is always such an abundance of excellent material to be had here, that at no time of the year can there be said to be an absolute dearth of operative cases. A large and comfortable operating room has been recently erected in the Presbyterian Hospital, and the cases there are at all times worth attending, as the operator takes great pains in explaining every step of the operation.

The four great hospitals of New York, viz.: the Presbyterian, New York, Roosevelt and Bellevue, are always objects of interest to the visiting doctors from out of town.

Dr. P. F. Munde, Professor of Gynecology at the New York Polyclinic, related to his class the history of a case of sufficient interest to repeat here. The patient was a married woman, 28 years of age, who was attended in her confinement by a midwife. This midwife gave the patient some medicine which produced severe uterine contractions, with the child occupying the transverse position. The visitor grew frightened and sent for a physician, who, when he came, found the head in the pelvic cavity, and delivered by the forceps. After the delivery of the child, he put his hand into the vagina and it went right into the abdominal cavity, the external os being firmly closed. Twelve hours later, Dr. Munde saw the patient, and on examination, he found an enormous rent in the posterior vaginal wall, through which his hand readily entered the abdominal cavity. He put iodoform gauze into the vagina, and placed a drainage tube in the peritoneal cavity through the rent in the vaginal wall, and waited till the next day to sew up the tear. When he again called, the temperature was 104 deg. F., and the pulse quick and small, so he did nothing. The drain was kept in a few days longer, and an offensive discharge flowed out. The vagina was irrigated gently, and the bowels moved by Rochelle salts. The rent in the posterior vaginal wall closed up spontaneously. Dr. Munde said he had seen a dozen such cases of tear of the vaginal vault, the child having escaped into the peritoneal cavity, but they all died. The point about the treatment of the case he wished to emphasize, was not to do too much.

